# Psychospiritual effects of visitation on hospitalized children

**DOI:** 10.1590/1806-9282.20250542

**Published:** 2025-09-19

**Authors:** Hüseyin Çaksen

**Affiliations:** 1 Necmettin Erbakan University, Faculty of Medicine, Department of Pediatrics, Divisions of Pediatric Neurology, Genetics, and Behavioral-Developmental Pediatrics – Konya, Türkiye.

Dear Editor,

Hospitalization is traumatic, anxious-inducing, and stressful for children. Common hospital stressors include fear of injury, fear of needles and pain, loss of control, separation from family and friends, and fear of death^1^. A recent study showed that 64% of hospitalized children had moderate anxiety, 22% had high anxiety, and 14% had low anxiety levels^1^. Distress of hospitalized children is evident when children are unaccompanied in the hospital and left to experience the stressors of hospitalization alone^2^.

Visiting the sick, a culture that strengthens solidarity and compassion among people, has existed in many societies around the world for thousands of years. Visiting the sick is intended to comfort and even aid in their healing. However, today, visiting the sick has decreased because social isolation and loneliness are widespread in many societies. In this article, we discuss the psychospiritual effects of visitation on hospitalized children in order to draw attention to the importance of visitation, overlooked by many health professionals. The term "psychospiritual" has entered psychological and religious discourse as a loose designation for the integration of the psychological and the spiritual. As a broad term, it can denote a variety of positions between psychology and spirituality: a supplementation, integration, identification, or conflation of the two fields^3^.

The hospital and the disease are stressors for children and caregivers, since stress can interfere with the normal development of young patients, affecting them in the long term. Admitting a child to the hospital means interrupting his or her normal daily life and changing the environment that is familiar to him or her^4^. Long-term hospitalization emotionally impacts any patient, especially children, and is defined as a long period of time during which the patient is hospitalized and experiences isolation from his or her family, friends, and home^1^. Therefore, hospitalized children want to be in touch with their family, relatives, and friends and expect their loved ones to come to the hospital. Most children can handle visits just fine if they are given age-appropriate information and support^5^. Visiting hospitalized children does not pose an increased risk of infection if hygiene requirements are met^6,7^. Flexible visiting policies lead to greater patient satisfaction with care and to positive impacts for both patients and families, and these stakeholders have clear preferences for open/flexible policies^8^.

There are sparse studies about the psychospiritual effects of visitation on hospitalized children in the literature. Yang et al.^9^ reported that the use of videoconferencing by some hospitalized children and families to conduct virtual visits with family and friends outside of the hospital was associated with a greater reduction in stress during hospitalization than those who did not use videoconferencing. In another study, participants reported that family separation during pediatric hospitalization was very difficult, and use of videophones mitigated these effects through decreasing feelings of isolation and anxiety, and increasing feelings of connection between family members^10^. On the other hand, a systematic review showed that visiting hospitalized family members enabled children/adolescents to better understand the reality and to preserve their relationships with family members^11^.

Visiting the sick is a recommended philanthropic deed in different cultures and religions, including Christianity, Judaism, and Islam, and is considered an aspect of benevolence and a work of mercy^12^. Inquiring after their health and visiting the sick—on the condition it does not tax them—is Sunnah and also atonement for sins. There is a hadith that says, "Receive the prayers of the sick, for they are acceptable." To look after the sick, especially if they are relations, or parents, in particular, is important worship, yielding a significant reward. To please a sick person's heart and console him is a sort of significant almsgiving. Yes, pleasures are experienced at the time of illness that arise from the kindness, pity, and compassion of those around, are most pleasant and agreeable, and reduce the pains of illness to nothing^13^.

Agreeably, 1 of 10 recommendations for child-friendly visiting policies in critical care is the provision of psychosocial support through psychologists or spiritual counsellors^5^. However, spiritual support services are either absent or limited in many developing countries, including Türkiye^14^. Chaplain services were utilized in 5% of admissions in a series including children with chronic, non-cancer diseases. Utilization was similar between religiously affiliated patients and unaffiliated patients. Christian patients demonstrated similar utilization as non-Christian patients. Utilization was significantly higher among patients with a length of stay >2 days, compared to those with a length of stay ≤2 days^15^. Donohue et al.^16^ reported that 42% of parents of hospitalized children requested a chaplain visit. Parents felt that chaplains provided religious and secular services, including family support and comfort, help with decision-making, medical terminology, and advocacy. Chaplains helped most parents maintain hope and reduce stress. Notably, 75% of parents viewed chaplains as a member of the healthcare team; 38% reported that chaplains helped medical personnel understand their preferences for care and communication. Most parents (66%) felt that hospital chaplaincy increased their satisfaction with hospital care^16^.

In conclusion, illness and hospitalization are traumatic, anxiety-provoking, and can lead to transient or long-term behavioral and psychological difficulties in children^17^. Limited studies have shown that visitations are beneficial for hospitalized children and their families. We think that visitations have positive effects on the physical, mental, social, spiritual, and religious health of children and their parents/caregivers and even visitors because the relationships between these five dimensions of health is very close, often intertwined, profound, and symbiotic^18^. We recommend that comprehensive studies including chaplains should be performed about this subject in populations with different beliefs and sociocultural structures. These studies will both fill the gap in the literature and guide healthcare professionals.

## Data Availability

The datasets generated and/or analyzed during the current study are available from the corresponding author upon reasonable request.
